# Determining the depth of meditation through frontal alpha asymmetry

**DOI:** 10.3389/fnhum.2025.1576642

**Published:** 2025-08-29

**Authors:** Dwivedi Krishna, Deepeshwar Singh, N. K. Manjunath

**Affiliations:** ^1^Swami Vivekananda Yoga Anusandhana Samsthana, Bangalore, India; ^2^Babasaheb Bhimrao Ambedkar University, Lucknow, India

**Keywords:** frontal alpha asymmetry, electroencephalogram, heartfulness meditation, depth of meditation, Visual Analogue Scale

## Abstract

**Background:**

Electroencephalogram (EEG) alpha asymmetry has become a pivotal area of research for understanding functional hemispheric differences in neuroscience. To the best of our knowledge, the relationship between frontal alpha asymmetry (FAA) and the depth of meditation has yet to be thoroughly examined. To address this gap, the present cross-sectional study was conducted to explore the meditative states of long-term meditators and non-meditators.

**Methods:**

This study examined 26 long-term heartfulness meditation practitioners (LTM) and 33 non-meditators (NM), aged 30 to 45 years. Frontal EEG activity was employed to assess frontal alpha asymmetry (FAA), while self-reported measures, including the Meditation Depth Questionnaire (MEDEQ) and the Visual Analogue Scale (VAS), were used to evaluate the depth of meditation.

**Results:**

The results demonstrated significant differences in self-reported meditation depth between the long-term meditators and non-meditators, as shown through MEDEQ and VAS assessments. Notably, the FAA findings exhibited distinct interaction effects that highlight variations between the two groups. Furthermore, a positive correlation was established between FAA and the depth of meditation, supporting the notion that EEG patterns are reflective of self-reported meditative experiences.

**Conclusion:**

The findings suggest that heartfulness meditation may modulate FAA patterns in practitioners, which could be linked to enhanced emotional balance.

## 1 Introduction

Meditation is an ancient practice with deep roots in spiritual and philosophical traditions across various cultures. It has recently garnered significant attention for its potential benefits to both physical and mental health. The practice spans a diverse array of methods from mindfulness meditation and Transcendental Meditation to Yoga Nidra, each with unique characteristics but sharing the common goal of cultivating a state of focused attention, heightened awareness, and inner peace (Leckie, 2001; Ravi Shankar et al., 2014; Huberty et al., 2019).

The resurgence of interest in meditation over the past few decades is largely due to its perceived benefits, which range from stress reduction and improved concentration to enhanced emotional regulation and cognitive function. In our fast-paced and stress-laden world, individuals are increasingly turning to meditation as a tool for managing mental health, enhancing well-being, and fostering personal growth (Jamil et al., 2023). While the subjective experiences of meditation practitioners have been extensively documented, the objective measurement of meditation depth and its corresponding neurobiological effects remains a complex and challenging area of research.

In addition to neuroimaging, electroencephalography (EEG) has emerged as a valuable tool for investigating the neurophysiological underpinnings of meditation. EEG is a non-invasive technique that measures electrical activity generated by the brain, providing insights into the dynamic processes underlying various mental states. One of the key aspects of EEG research in meditation is the analysis of brainwave patterns, particularly in the alpha frequency band (8–12 Hz). Alpha waves are typically associated with a state of relaxed wakefulness and are prominent during periods of inner focus and reduced sensory input. Studies have shown that meditation can significantly influence alpha activity, particularly in the frontal lobes, which are crucial for cognitive functions such as attention, decision-making, and emotion regulation (Katyal and Goldin, 2021). An EEG measure that has garnered attention in the context of meditation is frontal alpha asymmetry (FAA). FAA refers to the difference in alpha power between the left and right frontal lobes, with different patterns of asymmetry being associated with various emotional and cognitive states. These associations have led researchers to propose that the FAA could serve as a potential neural signature for emotional states and individual differences in emotional reactivity. Research, particularly by Davidson and colleagues, demonstrated that the greater left frontal activation is consistently associated with positive emotions, approach-related behaviors, and adaptive emotional regulation, while greater right-sided alpha power is linked to withdrawal motivation and negative affect (Davidson, 2004; Harmon-Jones and Gable, 2018). These findings provide a theoretical basis for expecting long-term meditators, particularly those practicing techniques that foster positive affect and inward absorption, to exhibit greater left-sided FAA compared to non-meditators.

Given the established role of emotion regulation in meditation, it is plausible that FAA could also be related to the depth of meditative experience. A deeper meditative state is characterized by heightened focus and reduced emotional reactivity, which might be reflected in changes in FAA. However, while previous studies have documented changes in EEG patterns during meditation (Bauer et al., 2022; Krishna et al., 2022), the specific relationship between FAA and meditation depth remains largely unexplored. Moreover, the neural mechanisms linking FAA to the depth of meditative experience are not yet fully understood, presenting an intriguing area for further research.

While several meditation styles have been studied in relation to FAA. The most notable mindfulness, focused attention, and open monitoring practice, heartfulness meditation (HM), introduces a unique dimension through the use of Pranahuti (yogic transmission), a non-verbal subtle energy said to facilitate deeper meditative absorption. Unlike traditional practices that rely solely on volitional attentional control, HM integrates a receptive meditative mode, potentially engaging distinct neurophysiological mechanisms. Preliminary studies suggest that HM may foster deeper states of relaxation and self-expansion, which could lead to unique FAA patterns, particularly during transmission states (Arya et al., 2018; van’t Westeinde and Patel, 2022). Thus, this study aims to examine whether HM elicits FAA patterns that differ from those found in other meditation modalities, thereby extending the current understanding of meditation-related neural signatures.

In an effort to address these gaps, the present study sought to comprehensively investigate the relationship between FAA and self-reported measures of meditation depth. The study employed EEG recordings to measure frontal alpha asymmetry in experienced meditators during meditation sessions of varying depths. All participants underwent the components of heartfulness meditation, including baseline, meditation, transmission, and post, to examine changes in FAA in response to different levels of meditative engagement. Additionally, self-report measures of meditation depth and visual analog scales were collected to correlate with the EEG data, providing a more holistic understanding of the relationship between subjective experiences and objective neurophysiological markers.

## 2 Methods and materials

### 2.1 Participants

The current cross-sectional study recruited 59 volunteers, aged 30 to 45 years were from South India. Long-term heartfulness meditation practitioners (LTM; *n* = 26), having over 5 years of experience with an average of 1 h of HM practice daily, and non-meditators (NM; *n* = 33) with no prior meditation experience participated in the present study. *A priori* power analysis was conducted using G*Power 3.1 for an independent-samples *t*-test (dependent means, standard deviation, two-tailed). According to a previous study, effect size (Cohen’s *d* = 0.7), a significance level of *α* = 0.05 (two-tailed), and power = 0.80, a total sample size of 56 participants (28 per group) was required (Alyan et al., 2021). EEG data were collected during the HM session and self-reported scales were obtained after the HM session. All participants reported no visual, auditory, or neuropsychiatric issues. Individuals with a history of drug or alcohol abuse were excluded. Healthy participants who showed their willingness to participate were included in the present study. None of the participants was engaged in any other ongoing research activity.

### 2.2 Experimental procedure

All participants provided demographic information, including age, gender, and year of HM experience. Group demographic characteristics are summarized in Table 1. Independent-samples *t*-tests (for age) and chi-square tests (for gender distribution) were conducted to assess between-group differences. No statistically significant differences were observed between long-term meditators (LTM) and non-meditators (NM) with respect to age (*p* > 0.05) or gender distribution (*p* > 0.05) as shown in Table 1. LTM reported their meditation experience in terms of duration (months) and the average length of each meditation session (hours). Self-reported scales, including MEDEQ and VAS, were administered after the EEG session, once participants had completed their heartfulness meditation (HM) session. The participant and the heartfulness meditation teacher (Guru) entered the meditation chamber and were seated comfortably approximately 3 to 4 m apart. Each participant then engaged in a 30-min heartfulness meditation session in the presence of the Guru, during which only the participants wore an EEG cap. Both groups (LTM and NM) were informed that the Guru would facilitate the transmission process. Participants in both the LTM and NM groups were instructed to adopt a comfortable cross-legged seated posture with their eyes closed for the entire duration of the HM session. An initial 5-min relaxation phase was provided, after which participants were guided to gently shift their awareness toward a subtle “source of light” located within the heart to begin the meditation practice. This meditative focus was maintained without interruption. After 10 min of meditation, a Guru initiated the transmission process, which continued for the subsequent 10 min. Thus, the active practice consisted of 10 min of meditation followed by 10 min of transmission. These sequences were counterbalanced between participants to prevent the sequential effect. Following the practice, participants were instructed to remain seated and relax for an additional 5 min.

**Table 1:** Demographic characteristics of participants.

Variable	LTM group (*n* = 26)	NM group (*n* = 33)	*p*-value
Age (years)	37.2 ± 4.1	36.8 ± 4.5	0.68
Gender (M/F)	14/12	18/15	0.89
Meditation experience (hours)	762 ± 611.01	—	—

### 2.3 Heartfulness meditation

Heartfulness meditation (HM) represents a contemporary adaptation of Raja Yoga structured to accommodate modern lifestyles. This practice integrates five principal components: relaxation, cleaning, prayer, meditation, and yogic transmission. The relaxation phase is designed to calm the body, prepare the mind through subtle suggestions, and facilitate the transfer of energy from the Earth to replace feelings of heaviness with lightness. Cleaning is conducted in the evening, which serves to purify the mind and body by removing the day’s accumulated impressions. Silent prayer before sleep strengthens the connection with the inner self and reaffirms life’s purpose. Meditation is ideally performed in the early morning, which centers on the inner light within the heart. A unique aspect of heartfulness is the concept of Pranahuti (yogic transmission), which is described as a “divine energy from the Source” that aids in human transformation. This technique was rediscovered by Shri Ram Chandra (Lalaji) and is a cornerstone of the practice. Yogic transmission refers to the subtle transfer of energy during meditation, which is thought to facilitate deeper states of meditation and emotional regulation. Unlike traditional meditation practices that rely primarily on the meditator’s focus and attention, yogic transmission is believed to enhance these states through a non-verbal, energetic influence, typically imparted by a Guru. This energy transfer is thought to stimulate greater receptivity in the meditator, promoting deeper introspection and facilitating the achievement of a calm, centered state more quickly and effectively. These deep meditative experiences may be reflected in distinct neural signatures, suggesting an objective basis for the reported psychological and physiological effects (van’t Westeinde and Patel, 2022).

### 2.4 Assessment

#### 2.4.1 Self-reported scale

##### 2.4.1.1 The Meditation Depth Questionnaire

The Meditation Depth Questionnaire (MEDEQ) was developed by Dr. Herald Piron in 2001, and used in the present study. It comprises 30 items across five distinct subdomains: (a) hindrance (MEDEQ-H) with six items (1, 4, 9, 12, 13, and 18) assesses boredom, impatience, and issues with motivation and concentration; (b) relaxation (MEDEQ-R) with three items (8, 17, and 20) emphasizes feelings of comfort, inner peace, and calmness; (c) personal-self (MEDEQ-PS) with seven items (2, 3, 5, 10, 14, 21, and 29) explores experiences of detachment from thoughts, deep understanding, and feeling centered; (d) transpersonal qualities (MEDEQ-TPQ) with eight items (11, 15, 19, 22, 24, 27, 28, and 30) includes emotions such as love, devotion, thankfulness, and connectedness; and (e) transpersonal-self (MEDEQ-TPS) with six items (6, 7, 16, 23, 25, and 26) reflects the disappearance of cognitive processes and the experience of unity with everything. Each item is rated on a scale from 0 (not at all) to 4 (very much), with responses summed to yield a total meditation depth score. The MEDEQ is widely used for its agnostic approach to various meditation practices and demonstrates high convergent validity (ranging from 0.64 to 0.93) and strong internal consistency, with a Cronbach’s alpha of *α* = 0.81 (Reggente et al., 2024).

##### 2.4.1.2 Visual Analogue Scale

The Visual Analogue Scale (VAS) is a subjective measurement tool often used to assess the intensity of experiences such as pain or other sensations. Developed in the early 20^th^ century by Hayes and Patterson, the VAS consists of a continuous line, typically 10 cm long, anchored by two extremes of the experience being measured. It allows participants to quantify their experiences by marking a point on a continuous line that represents their perception of a particular state or intensity of an experience. In meditation studies, the VAS is often employed to measure subjective aspects of meditation depth, such as relaxation, focus, or mindfulness. Participants were asked to rate their experience of meditation on a scale, with the low end of the scale (to the left) labeled as “no experience” or “very shallow” and the high end (to the right) as “deep experience” or “very profound.” The flexibility of the VAS allows it to capture the nuanced and individual nature of meditative experiences, providing researchers with a quantifiable measure that can be correlated with other physiological or psychological data. Its reliability and validity have been extensively studied across various contexts, including pain and mood assessment (Abend et al., 2014). For meditation measurement, VAS has shown good reliability, with consistent results across different sessions and populations.

##### 2.4.1.3 EEG recording

Participants sat comfortably with their eyes closed in a quiet, dimly lit room while EEG data were acquired for 30 min (baseline—5 min, meditation—10 min, transmission—10 min, and post—5 min). The EEG data were recorded using a 128-channel Geodesic EEG system (Electrical Geodesics Inc., United States). The Geodesic Sensor Net 128 channel layout to the international 10–20 system of electrode placement was used in the present study. The HydroCel Geodesic Sensor Nets, connected by thin rubber bands, were placed on the participants’ scalp after being soaked in saline solution for 10 min, as per the EGI manual. The reference electrode was placed at the top of the head (Cz), and a 50 Hz notch filter was used. Data were collected using Net-Station™ software (version 4.5.6). The impedance for each electrode was kept below 50 kΩ during the recording, as recommended by the manufacturer, and the EEG signals were sampled at 250 Hz.

### 2.5 EEG pre-processing

The EEG data were processed offline using the EEGLAB toolbox (version 2022). (Delorme and Makeig, 2004) A total of 20 electrodes (left frontal—10 and right frontal—10) were used to assess FAA (Krishna et al., 2022). The selected EEG data were filtered within an 8–12 Hz range. Raw EEG data were manually reviewed to remove artifacts, and independent component analysis (ICA) was used to eliminate additional artifacts such as eye movements, muscle activity, and head movements. A total of five participants [LTM, *n* = 2, female = 1; NM, *n* = 3, female = 2] were excluded from the analysis due to (i) ocular signals exceeding ± 100 mV and (ii) more than 20% of the signal having artifacts.

The data were exported in .mat format, and custom MATLAB scripts were used for FAA analysis. EEG signals were converted into the frequency domain using the Fast Fourier Transform (FFT) algorithm with a 2-s Hamming window and a 75% overlap between consecutive windows to reduce data loss. Alpha asymmetry frequency power (8–12 Hz) was calculated from the cleaned EEG data. FAA was computed by subtracting the natural log of the alpha power at the right electrode site from the natural log of the alpha power at the left electrode site. Negative values for FAA reflect greater alpha power in the left frontal cortex compared to the right. This greater alpha power is associated with positive emotional states and approach-oriented behaviors (Davidson, 2004). Conversely, positive values of FAA indicate greater right-sided alpha power, which is typically linked to withdrawal and negative emotional states. FAA values were normalized to a range of 0–1 using log-transformation to reduce the influence of outliers and ensure that the data met the assumptions of normality required for parametric analyses. The log-transformation approach has been widely used in EEG research to enhance the sensitivity and interpretability of asymmetry data (Harmon-Jones and Gable, 2018).

### 2.6 Data analysis

Statistical analysis was done using JASP (JASP 0.16) in Windows. The Shapiro–Wilk test and Levene’s test (both at *p* > 0.05) were used to verify normality and homogeneity of variance in noise-free data. Psychometric data were not normally distributed; hence, the Mann–Whitney *U*-test was used to determine whether there were statistically significant differences between the two independent groups’ means of MEDEQ and VAS. Repeated measures of ANOVA were chosen on FAA data due to its normal distribution, robustness in analyzing within-subject variations over multiple states (BS, Med, Trans, and Post), and sensitivity to detect interaction effects. Mauchly’s test indicated a violation of the sphericity assumption (*p* < 0.001); therefore, Greenhouse–Geisser corrections were applied. Additionally, Bonferroni corrections were applied to post-hoc comparisons to control for Type I error arising from multiple comparisons. Spearman’s correlation was used to check the relationship among the FAA, MEDEQ, and VAS in the LTM and NM groups separately. Statistical significance was considered at *p* < 0.05, and all *p*-values were two-sided.

## 3 Results

**Figure 1:**
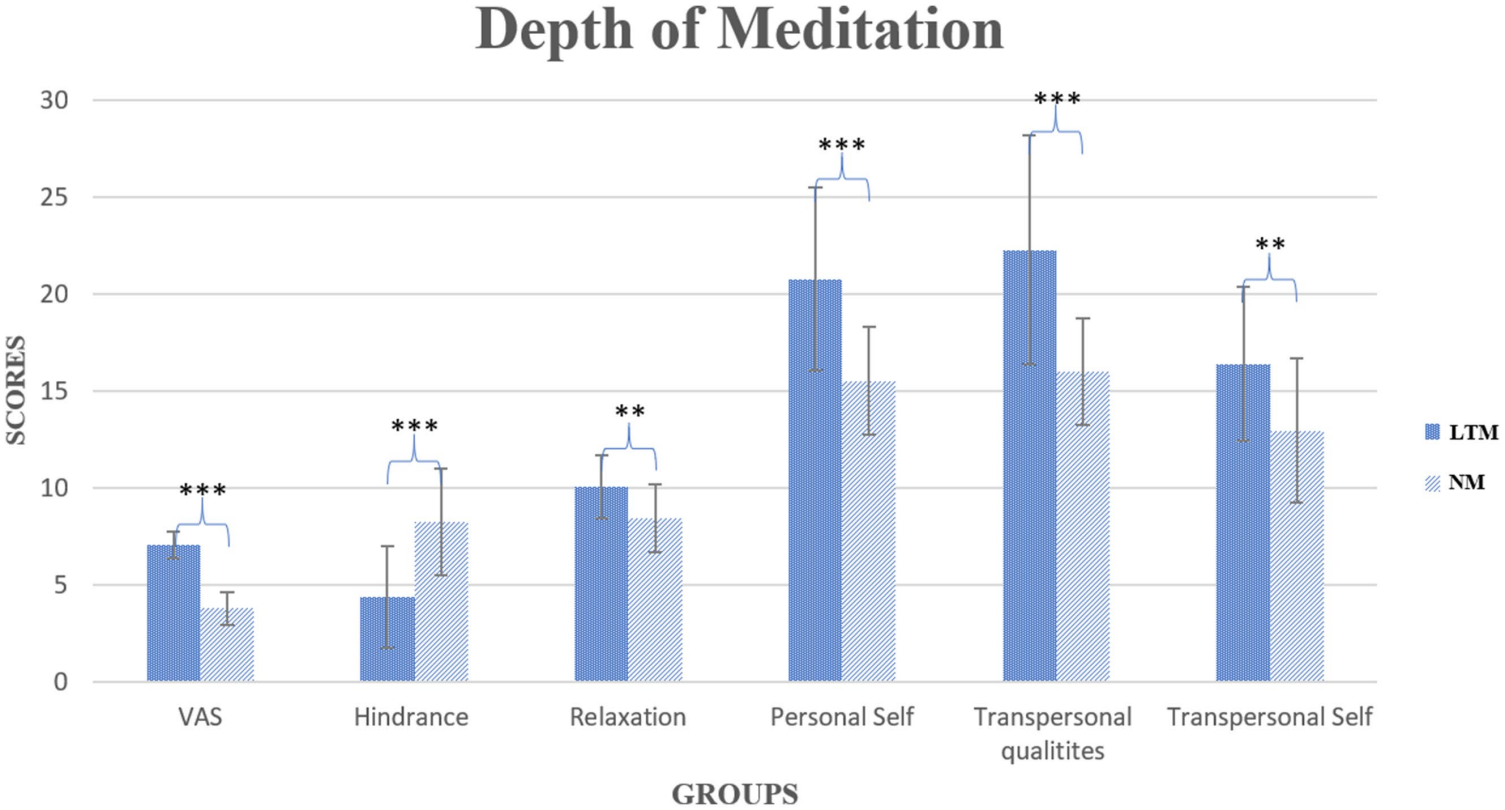
Graphical representation of the depth of meditation among LTM and NM. The bar graph shows the differences in groups (LTM and NM). The LTM group reported significant differences in all subjective variables. VAS, Visual Analogue Scale. **p < 0.01, ***p < 0.001, where * represents the between-group factor.

**Figure 2:**
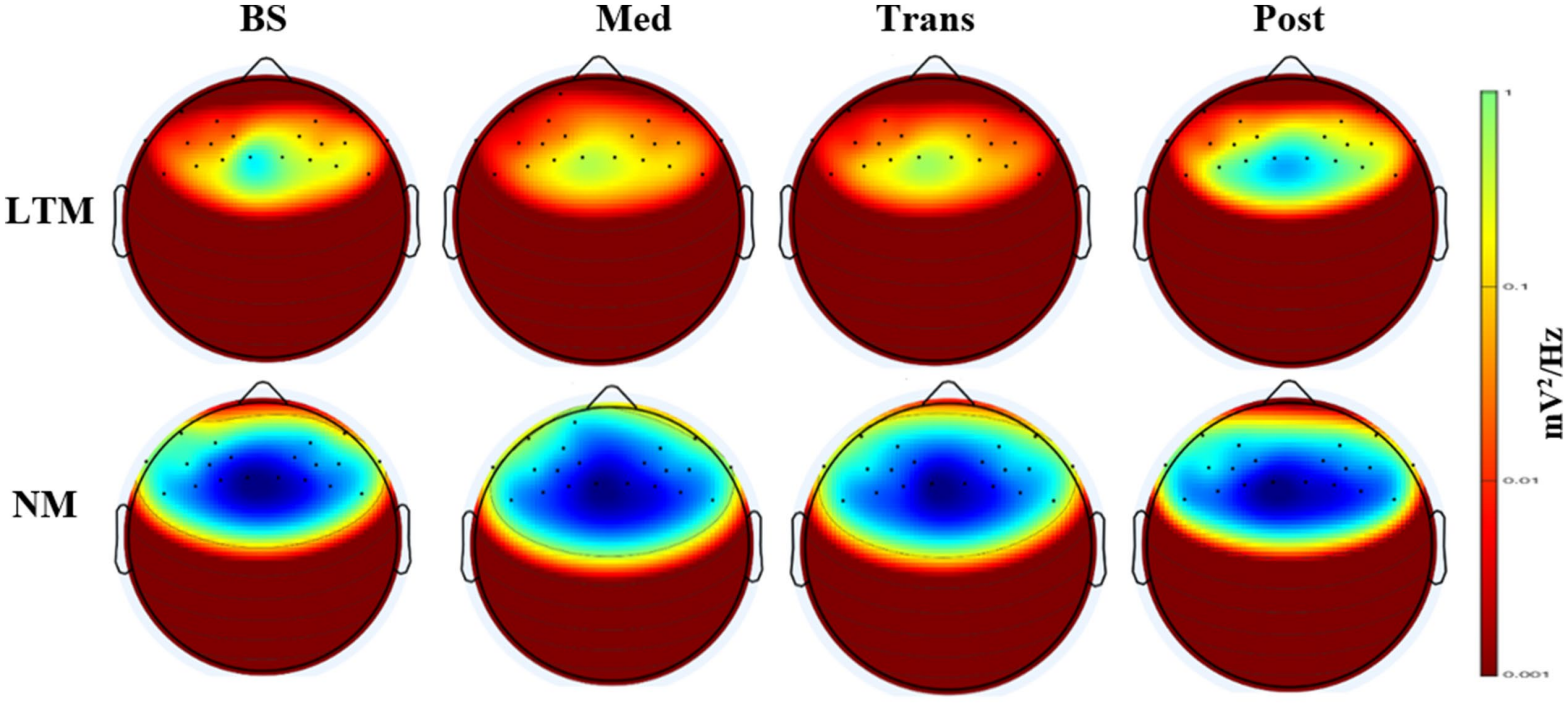
EEG topographical dynamics of alpha activity during heartfulness meditation.

The present study was conducted to assess the between-group comparisons between LTM and NM. The self-reported MEDEQ and VAS scores violated the assumption of normality. Therefore, the Mann–Whitney *U*-test was performed and showed significant differences among LTM versus NM groups in VAS (*U* = 2.00, *p* < 0.01), hindrance (*U* = 111, *p* < 0.001), relaxation (*U* = 182, *p* < 0.01), personal self (*U* = 127, *p* < 0.001), transpersonal qualities (*U* = 119, *p* < 0.001), and transpersonal self (*U* = 192, *p* < 0.01) shown in Figure 1. The repeated-measure ANOVA (RM-ANOVA) was performed to determine the effect of heartfulness meditation on LTM and NM. The results of the RM-ANOVA showed a significant main effect of group (*F*(1,52) = 10.473, *p* < 0.01, η^2^p = 0.168], but no significant main effect of state [*F*(2.22,115.53) = 0.098, *p* > 0.05, η^2^p = 0.002] and group × state interaction [F(2.22,115.53) = 0.187, *p* > 0.05, η^2^p = 0.004]. Post-hoc outcomes of the FAA are shown in Table 2. Moreover, a topographical comparison between LTM and NM of each state (baseline, meditation, transmission, and post) has been shown in Figure 2.

**Table 2:** Means and standard deviation of the FAA of the LTM and NM.

Groups/states	LTM (M ± SD)	NM (M ± SD)	*p*-value
BS	−0.139 ± 0.25	0.056 ± 0.31	*p* < 0.05
Med	−0.129 ± 0.21	0.039 ± 0.27	*p* < 0.05
Trans	−0.154 ± 0.23	0.063 ± 0.29	*p* < 0.01
Post	−0.132 ± 0.23	0.072 ± 0.28	*P* < 0.01

BS, baseline; Med, meditation; and Trans, transmission. Post-hoc outcomes of the FAA are presented in mean (M) and standard deviation (SD).

### 3.1 Correlation analysis

**Figure 3:**
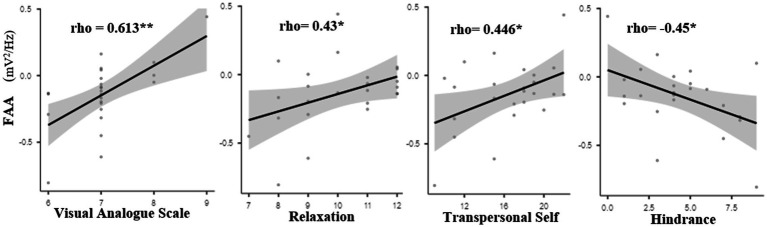
Relationship between frontal alpha asymmetry during transmission and the depth of meditation. Spearman correlation (*ρ*) between frontal alpha asymmetry (FAA) and self-reported VAS scores for depth of meditation during the transmission state in the long-term meditator (LTM) group. A significant positive correlation was observed. *p < 0.05, **p < 0.01.

Spearman’s correlation coefficient analysis of the FAA was conducted separately for both groups in relation to the transmission phase of HM practice. No significant correlations were observed among the variables in the NM group. In contrast, the LTM group demonstrated a significant positive correlation between FAA during Transmission and VAS (*ρ* = 0.613, *p* < 0.01), relaxation (*ρ* = 0.430, *p* < 0.05), and Transpersonal Self (*ρ* = 0.446, *p* < 0.05), along with a significant negative correlation with Hindrance (*ρ* = −0.450, *p* < 0.05). The correlation graph is presented in Figure 3.

## 4 Discussion

Meditation practices have been extensively studied for their potential benefits on physical and psychological well-being. Similarly, heartfulness meditation is a holistic practice that has garnered interest for its potential to reduce stress, induce relaxation, and improve overall mental health (Thakur et al., 2023). The outcomes of the present study revealed significant differences between LTM and NM groups across the VAS and MEDEQ scales. The LTM group reported higher levels of subjective experiences through VAS after meditation compared to non-meditators. A higher VAS score during meditation indicates that the individual perceives a greater depth of meditation, which often corresponds to measurable physiological, neurocognitive, and psychological changes linked to relaxation, awareness, and altered states of consciousness. This finding aligns with previous research that suggests meditation practices mitigate ruminative thoughts, resulting in improved awareness, mindfulness, and relaxation (Jain et al., 2007).

The present study showed significant differences in the depth of meditation, including psychological hindrance, relaxation, personal self, transpersonal qualities, and transpersonal self among LTM. This reduction in psychological hindrances may be attributed to the ability of practice to lower stress hormones (Goyal et al., 2014). LTM demonstrated marked improvements in relaxation, suggesting that regular practice can facilitate greater inner calmness and a reduction in mental clutter. It suggests that HM may facilitate such transpersonal experiences, which are linked to enhanced spiritual well-being and a deeper sense of meaning in life. Transpersonal experiences are often described as states of consciousness that transcend the individual ego and connect one to a universal consciousness. This could be one of the key differentiators between meditation practitioners and non-meditators that provides a potential explanation for the psychological benefits associated with long-term meditation practice. Heartfulness meditation facilitates personal self-reflection that may allow practitioners to strengthen their connection with their inner self and achieve a more balanced state of mind (van’t Westeinde and Patel, 2022). This self-awareness may be crucial for personal development and understanding one’s emotions. Potentially fostering transpersonal qualities that reflect a deeper sense of interconnectedness with oneself and others. Moreover, heartfulness meditation nurtures the transpersonal self by facilitating spiritual growth and self-transcendence that helps individuals move beyond the ego and fosters empathy and compassion.

Frontal alpha asymmetry (FAA) is a neurophysiological marker commonly associated with emotional and cognitive processes, which is characterized by the relative power of alpha waves in the left vs. right frontal regions of the brain. The outcomes of the present study showed significantly higher FAA among the LTM than the NM group at baseline, which suggests an enduring impact of HM on frontal cortex activity, leading to improved emotional regulation and approach-oriented behaviors (Wang et al., 2018). A scientific study reported that regular meditation has been shown to induce trait-like shifts in brain function with increased left-sided FAA, better emotional regulation, and approach-oriented behaviors (Deng et al., 2021). Alternatively, individuals with a naturally left-dominant FAA may find it easier to relax, thus being more inclined to engage in meditation practices (Brown et al., 2022). In this case, the FAA may reflect an individual predisposition to engage in practices suggesting that baseline differences could be driven by pre-existing emotional tendencies. Conversely, the NM group demonstrated higher right-sided cortical activity, which indicates the withdrawal of motivation or behaviors. This outcome is consistent with prior literature demonstrating that resting FAA in non-meditators often varies, with many healthy individuals exhibiting right-sided FAA reflecting typical individual differences in emotional reactivity and affective style (Harmon-Jones and Gable, 2018). Research supports that the increased left-sided frontal alpha activity in LTMs reflects the relationship between deep meditation and improved emotional stability and relaxation (Lee et al., 2015). This shift reflects a favorable emotional balance that facilitates deeper concentration and mindfulness, which are key aspects of deep meditation. Research has demonstrated that regular meditation alters brain regions associated with attention, self-awareness, and emotional regulation (Krishna et al., 2022). Neuroimaging studies on long-term meditators report changes in gray matter density in the prefrontal cortex, insula, and hippocampus regions critical for higher-order cognitive functions and emotional processing (Babu et al., 2020). Meditation practices also alter the neural activity of the brain, leading to more balanced frontal alpha asymmetry (Barnhofer et al., 2007). Additionally, studies of functional connectivity reveal that meditation enhances communication between brain networks involved in cognitive control and emotional processing, which underlies improvements in attention, emotional stability, and mental resilience (Lee et al., 2015). Emerging evidence aligns with previous studies suggesting that the left insula and cingulate cortex are associated with parasympathetic regulation and the right insula and cingulate cortex are linked to sympathetic regulation (Davidson, 2004). It has been shown that HM has a positive effect on parasympathetic modulation (Arya et al., 2018), which reflects that HM plays a crucial role in shifting towards a more relaxed and contributing to a balanced emotional state.

One unique aspect of heartfulness meditation is the practice of *Pranahuti* or yogic transmission, where practitioners believe they receive spiritual energy that supports their meditation. Yogic transmission was distinguished from the meditation component itself by its role in facilitating deeper meditative states through the subtle transfer of energy. This energy transfer is theorized to stimulate greater emotional clarity, attentional focus, and self-regulation in meditators. The current study observed higher FAA in LTM during transmission. These changes may reflect the enhanced emotional regulation and focus facilitated by the transmission process. Although the neurophysiological mechanisms of yogic transmission are not yet fully elucidated, it is proposed that this practice activates brain circuits associated with emotional processing and attention regulation, potentially offering distinct effects compared to conventional meditation techniques (van’t Westeinde and Patel, 2022). However, a placebo or expectation effect may have also contributed to the enhanced neural responses observed during the transmission phase. Expectancy effects, often referred to as demand characteristics in psychological research (Klein et al., 2012), could have influenced the outcomes in the LTM group. Since LTMs are more familiar with heartfulness meditation (HM), their higher expectations and emotional responses to the Guru’s presence may have enhanced the perceived depth of meditation and associated physiological effects. Therefore, future research should incorporate more rigorous control conditions to more effectively isolate the effects of yogic transmission from potential expectancy effects.

Moreover, during the transmission state, the FAA showed a significant positive correlation with the depth of meditation VAS, relaxation, and transpersonal self. It suggests that the natural inclination of the mind towards introspection and emotional processing allows for a release of pent-up emotional states associated with negativity. The synergistic effects of cognitive control, relaxation, and the reception of pure consciousness during meditation may lead to a state of self-awareness and transcendent experiences. Furthermore, the HM approach emphasizes a passive, receptive state during meditation, encouraging practitioners to let go of distractions rather than actively suppress them. This may be one of the regions where HM practitioners experienced a normalized brain activity that facilitates emotional stability and adaptive coping mechanisms. Emerging research pointed out that long-term meditation practice may yield lasting changes in neural pathways that underlie emotional and cognitive functioning (Basso et al., 2019; Gupta et al., 2022). The neural plasticity of the brain allows it to reorganize and adapt to new emotional states, which can help stabilize the frontal alpha asymmetry in a favorable direction. Observations suggest that regular HM practitioners may experience protective effects against stress and anxiety (Krishna et al., 2022) that are reflected in their heightened frontal alpha asymmetry. Additionally, depth in meditation has been shown to influence the connectivity within neural networks, particularly within the prefrontal cortex and its interactions with other brain regions (van Leeuwen et al., 2012). It is reported that deeper meditative states lead to stronger interhemispheric communication and coordination, enhancing overall brain functionality and coherence (Krishna et al., 2022; Shrivastava et al., 2023).

In the present study, frontal alpha asymmetry showed a negative correlation with hindrance. It determines that heartfulness meditation optimizes neural inhibition, and improves emotional well-being and psychological resilience, thereby reducing various mental hindrances. These hindrances often manifest as stress, anxiety, and negative thought patterns, which can obstruct mental clarity and overall quality of life. The mechanisms underlying the effectiveness of HM in mitigating such hindrances primarily involve neurophysiological changes and emotional regulation strategies. Research indicates that engaging in HM promotes a state of relaxation and reduced arousal of the stress response system (Gupta et al., 2023; Thakur et al., 2023). During meditation, practitioners focus on their hearts and are guided to become more aware of their thoughts and feelings. This practice facilitates the development of mindfulness, which has been shown to decrease ruminative thoughts and emotional reactivity, thus lessening feelings of stress and anxiety that may hinder overall functioning. Moreover, yogic transmission is believed to enhance the meditative experience by providing a supportive and calm energy that helps participants navigate distracting thoughts and emotions. This process assists in the release of emotional baggage, further reducing mental clutter and allowing for clearer decision-making and engagement with the present moment. Hence, LTMs are able to achieve the depth of meditation in a shorter period of time.

The findings of this HM study show promising implications for its integration into therapeutic settings, particularly in mental health care. In this study, the HM group scored significantly lower on the hindrance subscale of the MEDEQ compared to the NM group, reflecting greater ease in emotional regulation. Additionally, the HM group demonstrated more favorable outcomes, including enhanced relaxation and transpersonal self-awareness. These results suggest that HM could serve as a valuable adjunct to conventional therapeutic approaches, such as cognitive behavioral therapy (CBT) and mindfulness-based interventions (MBIs). By incorporating structured meditation practices into treatment plans, clinicians can promote emotional regulation, resilience, and stress reduction in patients dealing with anxiety, depression, or burnout. Moreover, the unique aspect of *Pranahuti*, or yogic transmission, can facilitate deeper introspection and foster a sense of community in group therapy settings. This practice not only nurtures individual well-being but also encourages empathic connections among group members. With empirical evidence, therapists can leverage heartfulness meditation as a holistic approach, addressing both psychological and physiological aspects of mental health, ultimately leading to improved patient outcomes and overall wellness.

This study has a few limitations that warrant consideration. The sample size is relatively modest, which may limit the statistical power and generalizability of the findings. The meditators had over 5 years of HM experience with varying levels of experience. The Meditative Depth Questionnaires are based on self-reported data, which may have potential biases such as social desirability and subjective interpretation. Larger, more diverse samples and randomized designs in future studies are necessary to validate and extend these findings to broader populations. While the FAA provides valuable insights into emotional regulation, other EEG markers, such as theta or beta waves, may also play an important role in understanding emotional processing and meditation effects. Future research could examine the interplay of multiple EEG markers in relation to meditation practices and emotional states to provide a more comprehensive understanding of brain dynamics during meditation. Simultaneous EEG recordings from both the Guru and the meditators could provide a more direct assessment of the potential transmission of subtle energy during the meditation session. However, this study did not include such measurements, limiting our ability to clearly and objectively characterize the neural dynamics involved in the transmission process. Additionally, the inherently subtle and subjective nature of yogic transmission presents significant challenges for measurement using current neurophysiological methods. Future studies may consider incorporating simultaneous EEG or other neuroimaging techniques for both participants and may implement rigorous control conditions to better elucidate and validate the underlying mechanisms of this phenomenon. Moreover, studies are warranted to involve a novice participant sitting in the Guru’s position while LTMs undergo the transmission phase with their eyes closed, without the Guru being present. This may help evaluate the extent to which the observed effects are driven by the placebo or expectation effect.

However, the study does have several strengths. It explores an understudied area by assessing meditation depth using both neural signals (FAA) and subjective VAS and MEDEQ measures, providing a multidimensional perspective of the depth of meditation. The inclusion of highly experienced meditators allows for a deeper understanding of the long-term effects of HM. Finally, the study contributes to the growing body of research on the neural correlates of meditation, particularly in the context of heart-based practices.

## 5 Conclusion

In summary, heartfulness meditation serves as an effective intervention for reducing mental hindrances by enhancing mindfulness and promoting psychological resilience. Its structured approach not only aids individuals in navigating their internal landscapes but also equips them with strategies to better handle external stressors. HM cultivates a state of inner peace and resilience, and stimulates the brain regions responsible for emotional regulation and positive cognition. This meditation practice promotes deeper states of consciousness and facilitates a transformative journey toward self-realization and emotional well-being. The present study highlights the relationship between FAA and the depth of meditation in LTM. FAA values were significantly higher in LTMs compared to NM, suggesting that HM may enhance emotional regulation and cognitive processes. Hence, we may consider the FAA as a neural marker of emotional balance and meditative depth. The outcomes of the study may add to the growing evidence on the neurophysiological correlates of meditation. These findings underscore the potential of HM to promote emotional well-being and offer a valuable tool in mental health interventions. These shreds of evidence are consistent with the theoretical underpinnings of Heartfulness Meditation, which emphasizes achieving a balanced and harmonious state of mind through contemplating the light in the heart. Further research is warranted to understand its role in facilitating deeper states of consciousness and exploring underlying neural mechanisms.

## Data Availability

The original contributions presented in the study are included in the article/supplementary material, further inquiries can be directed to the corresponding author.
