# Correction: Determining the depth of meditation through frontal alpha asymmetry

**DOI:** 10.3389/fnhum.2025.1695594

**Published:** 2025-09-12

**Authors:** Dwivedi Krishna, Deepeshwar Singh, N. K. Manjunath

**Affiliations:** ^1^Swami Vivekananda Yoga Anusandhana Samsthana, Bangalore, India; ^2^Babasaheb Bhimrao Ambedkar University, Lucknow, India

**Keywords:** frontal alpha asymmetry, electroencephalogram, heartfulness meditation, depth of meditation, Visual Analogue Scale

The funder: Swami Vivekananda Yoga Anusandhana Samsthana (S-VYASA), Deemed to be University, Bengaluru was erroneously omitted.

The correct funding statement appears below:

The author(s) declare that financial support was received for the research and/or publication of this article. The current study was supported by the Institutional Seed Money Grant by Swami Vivekananda Yoga Anusandhana Samsthana (S-VYASA), Deemed to be University, Bengaluru.

The original version of this article has been updated.

